# Antennal grooming facilitates courtship performance in a group-living insect, the German cockroach *Blattella germanica*

**DOI:** 10.1038/s41598-019-39868-x

**Published:** 2019-02-27

**Authors:** Ayako Wada-Katsumata, Coby Schal

**Affiliations:** North Carolina State University, Department of Entomology and Plant Pathology and W.M. Keck Center for Behavioral Biology, Raleigh, North Carolina USA

## Abstract

The antennae of adult male German cockroaches detect a contact sex pheromone embedded in the female’s cuticular lipids. The female pheromone stimulates courtship behavior in males, notably a wing-raising (WR) display. Within aggregations, however, cuticular lipids are disseminated by contact among group members, including nymphs and adults of both sexes, and “contamination” of cockroaches with the cuticular lipids of another stage or sex may interfere with sex discrimination and disrupt courtship. We used behavioral observations, bioassays and chemical analysis to determine how males maintain their sensitivity to sex pheromone in aggregations. Males contaminated with female pheromone displayed lower courtship, because residual female pheromone on their antennae adapted their peripheral sensilla and habituated the central nervous system. Female pheromone that contaminated the male’s antennae also elicited courtship from other non-contaminated males, disrupting their sex discrimination in the group. However, antennal grooming effectively removed female pheromone from males’ antennae and maintained their chemosensory acuity and sexual discrimination among group members. Thus, grooming of the antennae and other sensory appendages is an important strategy to enhance sensory acuity, especially in group-living insects like the German cockroach.

## Introduction

Insect cuticular lipids, composed mainly of relatively non-volatile apolar lipids, cover the insect cuticle and serve as barrier that prevents desiccation and penetration of harmful agents including pathogens^1^. A major chemical group of cuticular lipids is the cuticular hydrocarbons (CHCs), synthesized in oenocytes and selectively transported to the cuticular surface^1^. In many insect species, CHCs and their derivatives function as semiochemicals, mediating various behaviors including mate-finding, courtship, aggregation, aggression, and recognition and discrimination of sex, host, colony members and fertility of individuals^2–6^.

**Figure 1:**
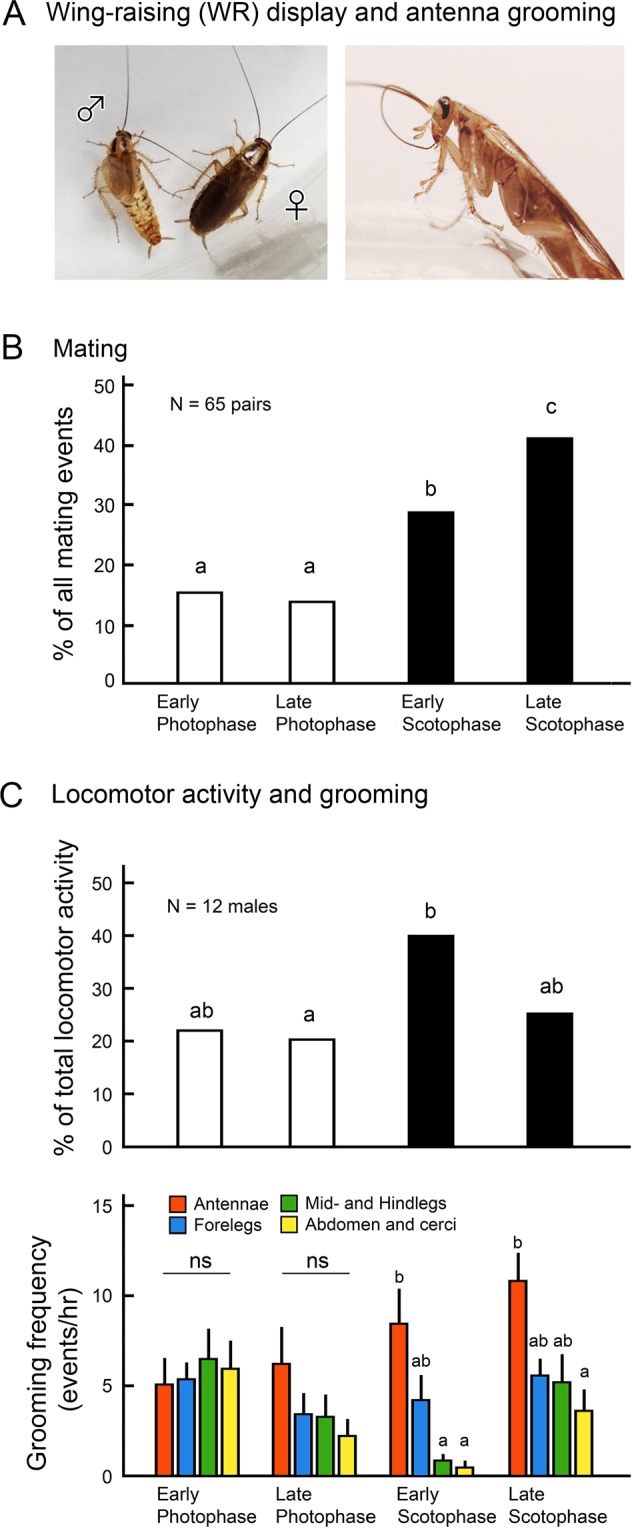
Wing-raising courtship display, mating and antenna grooming in male *B*. *germanica*. (**A**) Male wing-raising display to a female (Left) and antenna grooming (Right). (**B**) Distribution of mating events of 65 pairs of cockroaches over the photocycle. Different letters indicate significant differences (Chi-square test, *p* < 0.05). The highest frequency of mating was observed in late scotophase. (**C**) Upper, Locomotor activity of individual males. Different letters indicate significant differences (Chi-square test, *p* < 0.05). Bottom, Grooming frequencies of different body parts (mean ± SE). Different letters indicate significant differences (ANOVA with Tukey’s HSD, *p* < 0.05). Locomotor activity and antenna grooming frequency were significantly higher in the scotophase than in photophase.

Females of the German cockroach, *Blattella germanica*, use components of their cuticular lipids as contact sex pheromone that stimulates males to recognize and court the female^7^. Males are attracted to a volatile sex pheromone, blattellaquinone, emitted by sexually mature females^8^. Upon antennal contact with the female body and antennal “fencing”, the male’s antennae detect a female contact sex pheromone consisting of a complex blend of six components, with 3,11-dimethylnonacosan-2-one^9^ and 3,11-dimethylheptacosan-2-one^10^ being most abundant. Immediately, the male executes a wing-raising display (WR, Fig. 1A, SI Video 1), consisting of a turn away from the female while raising his wings to a vertical position. This display exposes a specialized tergal gland that offers a pre-nuptial gift to the female^11–13^. As the female palpates and feeds on the tergal secretion, the male extends his abdomen, grasps the female’s genitalia, and copulation follows^7^.

The German cockroach is a gregarious insect, and courtship is commonly observed in crowded aggregations that contain both sexes and various developmental stages in close proximity, frequently touching each other’s antennae to maintain group cohesion^14^. This group-living arrangement may cause inadvertent exchange of cuticular lipids among individuals, possibly compromising the effectiveness of the sensory system that makes sex recognition and discrimination possible. Since nuptial feeding never occurs without the WR display however, the male’s perception of the contact sex pheromone is extremely important and males are expected to evolve biochemical and behavioral mechanisms to enhance discrimination of the sexes.

Insects have evolved elaborate physiological mechanisms to bind hydrophobic ligands, transport them through the aqueous sensillar lymph, present specific ligands to neuronal receptors, and enzymatically metabolize pheromones and xenobiotics in the sensillar lymph^15^. But behavioral mechanisms for enhancing sensory acuity have not been examined. Nevertheless, the recognition that antennal grooming is important in eliminating environmental contaminants from insects^16–21^, and recent evidence that antennal grooming prevents a build-up of CHCs that can interfere with olfaction^22^, prompted us to consider this behavior in male-female interactions. First, we determined whether grooming frequency of German cockroach male antennae increases during behavioral activity, including courtship. Then, using chemical analysis and behavioral assays, we determined if female cuticular lipids are transferred to males within a group, and if grooming contributes to removal of the residual female pheromone from the contaminated male antennae, thus maintaining WR performance. We also tested if female cuticular pheromone on the male’s antennae causes peripheral sensory adaptation and/or central nervous system (CNS) habituation to the sex pheromone. We discuss the importance of grooming for chemosensory behaviors in group-living insects.

## Results

### Greater locomotor activity and CHC accumulation in scotophase

Mating, locomotor activity and antenna grooming occurred more in the scotophase than in the photophase (Fig. 1B). Mating events were significantly more common in the late than in early scotophase (41.5% and 29.2%, respectively) (Chi-square test, df = 3, χ^2^ = 17.621, *p* < 0.01), while the percentage of locomotor activity was more evenly distributed, but highest in early scotophase (39.3%) (Fig. 1C) (ANOVA, df = 3, *F* = 3.21, *p* = 0.032). The frequency of antenna grooming was significantly higher than that for other body parts during the scotophase, while no significant differences in the grooming frequency among body parts were observed in the photophase (Fig. 1C) (ANOVA and Tukey’s HSD: Early photophase, df = 3, *F* = 0.229, *p* = 0.876; Late photophase, df = 3, *F* = 1.535, *p* = 0.219; Early scotophase, df = 3, *F* = 9.27, *p* < 0.01; Late scotophase, df = 3, *F* = 3.13, *p* = 0.035). All tested pairs successfully mated.

**Figure 2:**
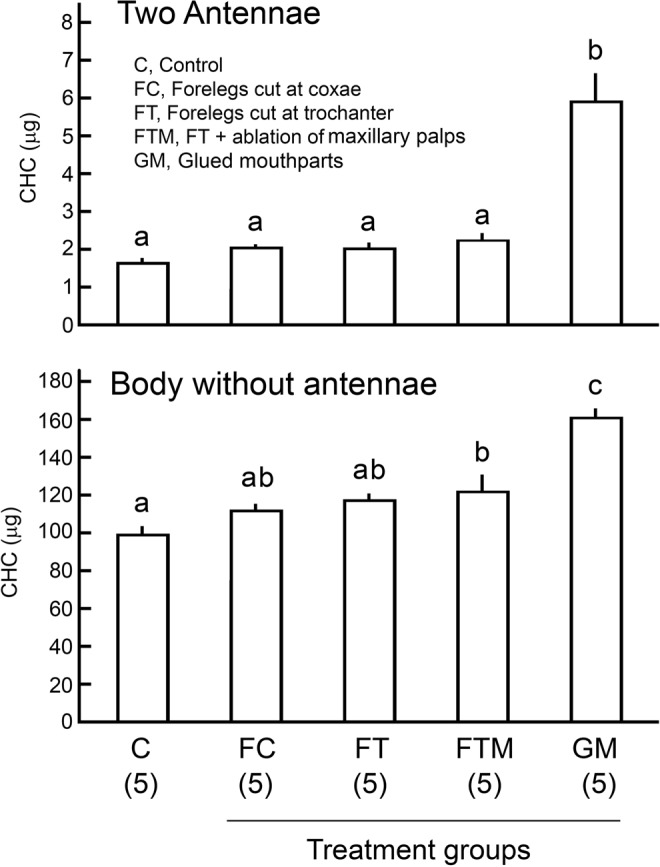
Amounts of cuticular hydrocarbons (CHCs) on *B*. *germanica* males prevented from grooming (mean ± SE). When the mouthparts were blocked with super glue, treated cockroaches could not remove accumulated CHCs from their antennae and body, indicating that the mouthparts are important in grooming. Insects were extracted after the various manipulations were performed. Different letters above the bars indicate significant differences (ANOVA with Tukey’s HSD, *p* < 0.01). Numbers in parentheses indicate number of insects tested.

**Figure 3:**
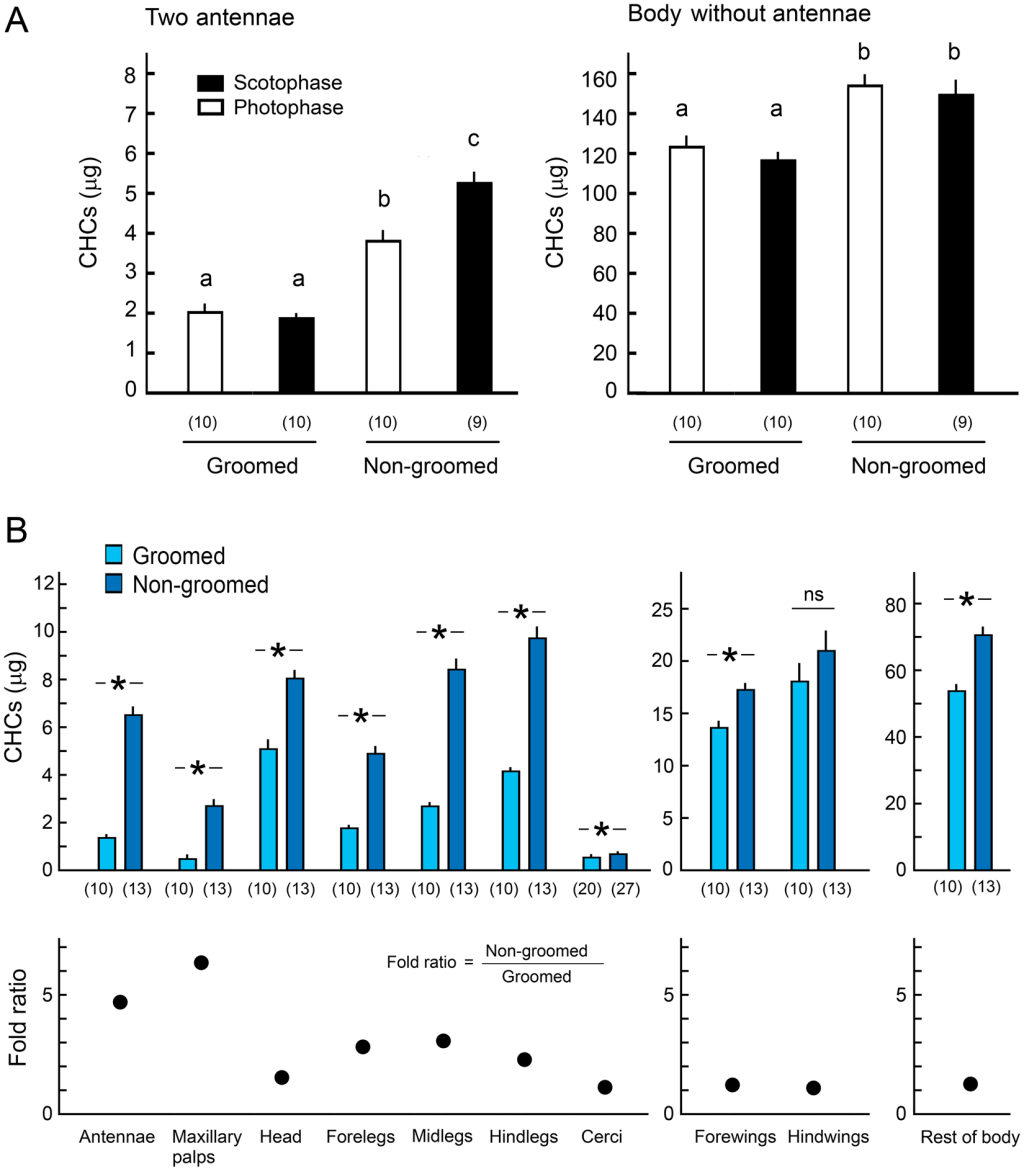
Accumulation of cuticular hydrocarbons (CHCs) on different body parts in freely grooming and non-groomed male *B*. *germanica*. (**A**) Amount of CHCs in scotophase and photophase (mean ± SE) extracted from antennae (left) and the rest of the body (right). Groomed, control freely grooming male; Non-groomed, a single male with glued mouth. Control males could groom, and maintained the same amount of CHCs in scotophase and photophase, while males that were prevented from grooming accumulated more CHCs on their antennae, particularly in the scotophase. Different letters above the bars indicate significant differences (ANOVA with Tukey’s HSD, *p* < 0.05). Numbers in parentheses indicate number of insects tested. (**B**) Upper, Amount of CHCs on different body parts (mean ± SE). All body parts except the hindwings accumulated significantly more CHCs when grooming was prevented. Asterisks indicate significant difference (see SI Table 1, Unpaired Student’s *t*-test, *p* < 0.05). Groomed, control freely grooming males; Non-groomed, treatment a single male with glued mouth (maxillary palps free) was immobilized in a pipette tip with only its head exposed out of the pipet tip; Specifically for the cerci, a male was put in the pipet tip with only its cerci exposed out of the pipet tip. Non-groomed males could not move or groom any of their body parts. Numbers in parentheses indicate number of tested insects. Bottom, Fold ratio of accumulated CHCs. Two pairs of chemosensory organs, the antennae and maxillary palps, accumulated relatively more CHCs than other body parts.

For evaluating CHC accumulation by chemical analysis, CHC amounts of non-groomed (treatment, grooming-prevented group) and groomed (control, freely-groomed group) insects were compared. The most effective method to prevent grooming was to glue the mouthparts using super glue (Fig. 2). When the mouthparts were blocked, treated cockroaches could not remove the accumulated CHCs from their antennae and body, indicating that the mouthparts are important in grooming. Other ablation treatments were not effective in preventing grooming, as evidenced by the accumulation of excess CHCs on the antennae and body (ANOVA with Tukey’s HSD: Antenna CHCs, df = 4, *F* = 25.05, *p* < 0.01; Body CHCs, df = 4, *F* = 20.96, *p* < 0.01). Figure 3A shows the total CHCs of groomed control group and the non-groomed group with glued mouthparts in the scotophase and photophase. The groomed cockroaches maintained the amount of CHCs on their antennae and body, in both the scotophase and photophase (Fig. 3A). However, preventing grooming resulted in significant accumulations of CHCs on the antennae and body, and the antennae accumulated significantly more CHCs in the scotophase than photophase (ANOVA and Tukey’s HSD: Antennae, df = 3, *F* = 61.23, *p* < 0.01; Body, df = 3, *F* = 19.31, *p* < 0.01).

Different body parts differentially accumulated CHCs in the non-groomed cockroaches (Fig. 3B). The amounts of CHCs on all body parts, except the hind wings, of males that were prevented from grooming, were significantly higher than in freely groomed insects (unpaired Student’s *t*-test: *p* < 0.05) (SI Table 1). Notably, the respective ratios of CHCs in non-groomed to groomed body parts were highest for two chemosensory appendages – antennae and maxillary palps – which accumulated 4.7- and 6.3-fold more CHCs in non-groomed insects. These results suggest that cockroaches actively refresh their cuticular lipids, particularly on sensory appendages, by a combination of grooming and CHC secretion in the scotophase, when they also engage in courtship behavior.

### Contamination of male antennae with female pheromone disrupts sexual discrimination

**Figure 4:**
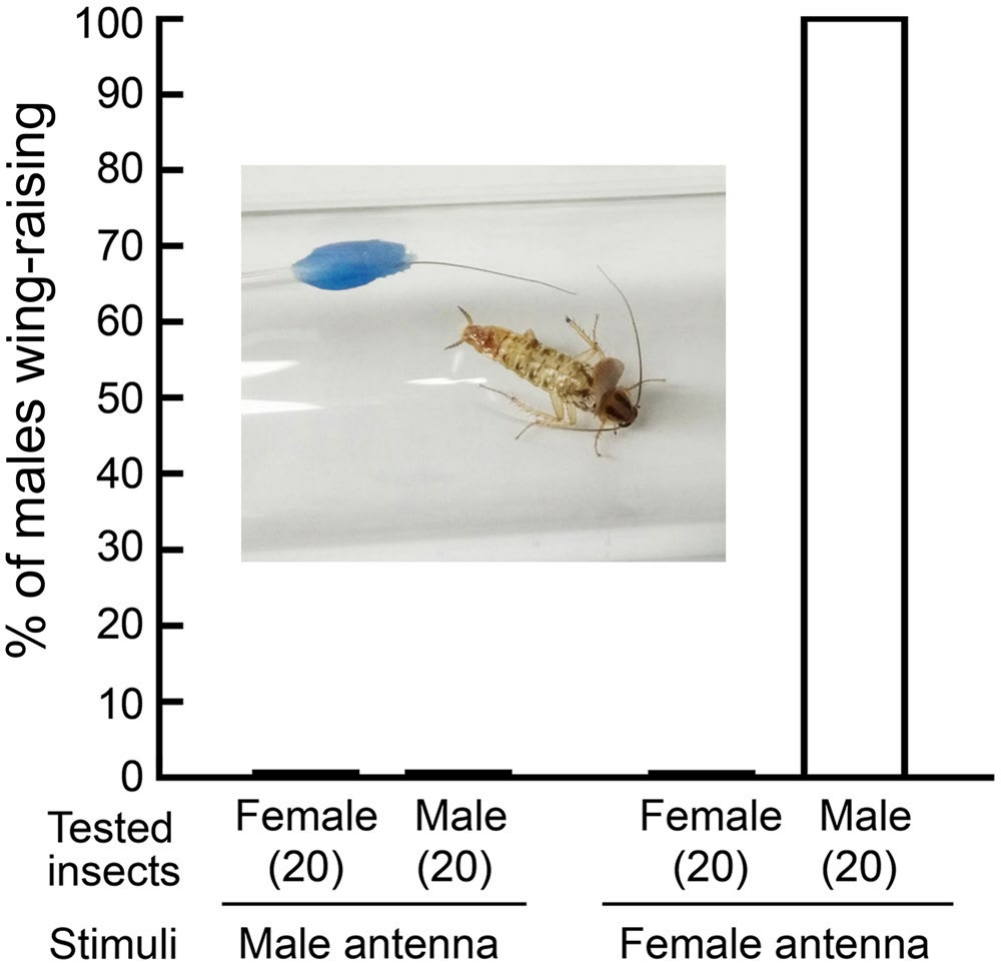
Context of the wing-raising (WR) display of male *B*. *germanica*. The photograph shows the WR response of a male to an isolated female antenna in the “antenna-on-a-stick” assay. All males showed WR responses upon stimulation with only a female antenna, as shown in the photo, because a contact sex pheromone is contained in the female’s cuticular lipids. Females did not respond with WR to the antennae of either males or females, indicating that the WR display is a male-specific courtship behavior. Numbers in parentheses indicate number of insects tested.

**Figure 5:**
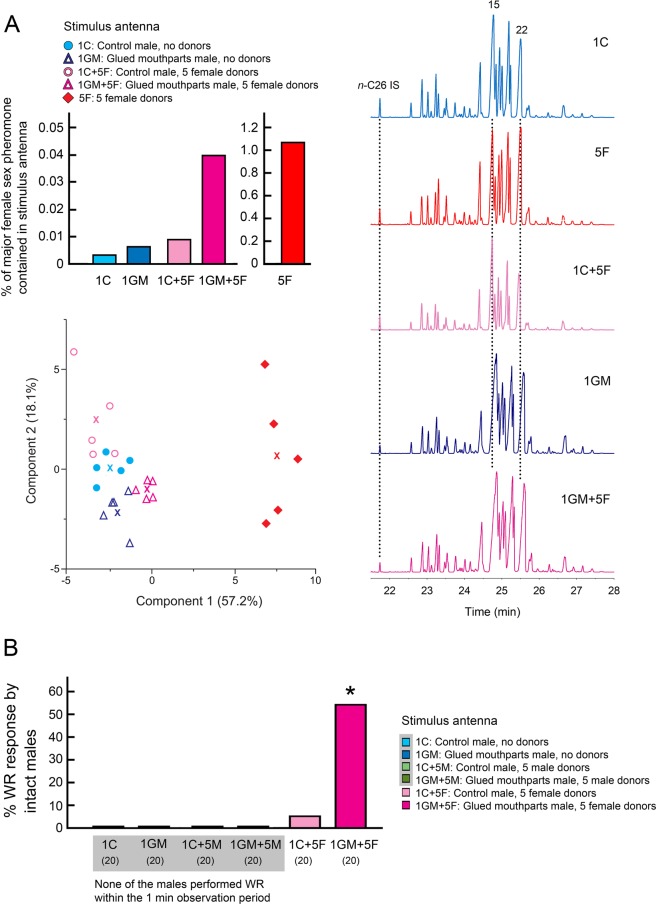
Female contact sex pheromone and CHCs contaminate male antennae in group living. (**A**) (upper left), Percentage of methyl ketone pheromone (3,11-dimethylnonacosan-2-one) in total CHCs per antenna in males and females. 1 C, Control male; 5 F, Females; 1 C + 5 F, Normal male housed with 5 females (1 C + 5 F); 1GM, Male with glued mouthparts; and 1GM + 5 F, Male with glued mouthparts housed with 5 females. Males that were prevented from grooming their antennae (with glued mouthparts) and housed with females (1GM + 5 F) accumulated significantly more female pheromone on their antennae than all other treatments of males (See SI Table 2). (A) (right), Gas chromatograms of CHCs from five treatment groups. The internal standard (IS) used in quantification (100 ng *n*-C26) is indicated, as well as two peaks that correspond to the CHCs in SI Table 3 and to a previous report^29^. Peak 15 (9-, 11-, 13-, and 15-methylnonacosane) is more common in normal males, whereas peak 22 (3,7-, 3,9-, and 3,11-dimethylnonacosane) serves as precursor to several components of the female contact sex pheromone, and is more common in normal females. (**A**) (bottom left), Principal components analysis (PCA) of the CHCs of five treatment groups. Different symbols indicate CHCs profiles of each replicate in different treatment groups (n = 5). X indicates the mean of each treatment group and symbols and colors correspond to the legend above. All male treatments differentiated from female CHCs. However, the CHCs of males with glued mouthparts that were housed with females (1GM + 5 F) were more similar to female CHCs. The results indicate that when a normal male was housed with five females, he removed the female CHCs that contaminated his antennae and maintained his own cuticular lipids profile. (**B**), Percentage courtship response (wing raising, WR) of intact males to male- and female-contaminated male antennae. When males were housed with five females and could not groom their antennae, their antennae were contaminated with female contact sex pheromone, disrupting sexual discrimination in normal intact males that were stimulated by the contaminated antenna. Asterisk indicates significant difference (Chi-square test, *p* < 0.05). Numbers in parentheses indicate number of insects tested.

Intact males that contacted a female antenna responded with a WR display, but never directed WR toward sexually mature male antennae (Fig. 4). The WR latency to a female antenna was 2.7 ± 0.15 sec (mean ± SE). Females never execute the WR display when stimulated with either female or male antenna, showing that WR is a male-specific behavior executed exclusively with high sensitivity and selectivity only during courtship. The major female sex pheromone component, 3,11-dimethylnonacosan-2-one (methyl ketone) is contained in the female’s cuticular lipids (10.9 ng methyl ketone in 1.02 µg CHCs per antenna) and the antennae of males contained barely detectable amounts of the methyl ketone (0.027 ng in 0.91 µg CHCs per antenna) (1 C and 5 F in SI Table 2). SI Table 2 shows that each antenna of males prevented from antenna grooming (1GM), contained 0.15 ng methyl ketone in 2.54 µg CHCs. Normal males that were housed with females (1 C + 5 F), and could groom their antennae, contained 0.065 ng in 0.75 µg CHCs per antenna, whereas males with glued mouthparts (no antenna grooming) housed with females (1GM + 5 F) contained 0.901 ng in 2.26 µg CHCs per antenna. Significantly higher amounts of methyl ketone were detected in 1GM + 5 F than in 1GM + 5 F males (ANOVA, *F*_3,16_ = 43.6008, *P* < 0.0001, SI Table 2). These results indicate that when grooming behavior was disabled, males could not remove female sex pheromone contamination from their antennae. The percentages of methyl ketone pheromone in CHCs per antenna of males and females are shown in Fig. 5A (upper left). Additionally, we compared the CHC profiles of various treatments to those of males and females. One CHC peak (Peak 22), that contains 3,7-, 3,9-, and 3,11-dimethylnonacosane, also serves as precursor to several components of the female contact sex pheromone, and is enriched in females, whereas 9-, 11-, 13-, and 15-methylnonacosane (Peak 15) is more abundant in males (Fig. 5A (right) and SI Table 3). The antennal CHCs of 1GM + 5 F contained relatively more of peak 22 than other males. Principal component analysis of the CHCs showed that the CHCs of all male treatments differentiated from female CHCs (Fig. 5A, bottom left). However, the CHCs of males with glued mouthparts that were housed with females (1GM + 5 F) were more similar to female CHCs. The results indicate that when a male is housed for 24 hrs with five females, he effectively removes female cuticular lipids that contaminate his antennae and maintains his own cuticular lipid profile. Moreover, a comparison of the CHCs of various male treatments revealed that grouped males (1 C + 5 F and 1GM + 5 F) tended to have lower amounts of CHCs per antenna than non-grouped males (1 C and 1GM, respectively) (SI Table 2). These results suggest that the amount of CHCs on the antennae may be affected by behavioral activity including the frequency and intensity of grooming in a group vs. in isolation.

Bioassays using antennae from males of different treatment group (Fig. 5B) to stimulate male courtship revealed that when we glued the mouthparts of female-contaminated males (1GM + 5 F), 55% of the intact test males courted the contaminated male antennae with a latency of 14.8 ± 4.91 sec. The contaminated antenna from normal males capable of grooming (1 C + 5 F) could elicit WR in only 5% (1 of 20) of intact test males with a latency of 17 sec. The male antennae in all other treatment groups failed to elicit WR responses from the test males (Chi-square test, df = 5, χ^2^ = 54.444, *p* < 0.01). These results indicate that antenna grooming is a critical behavior for removing female pheromone from the male’s antennae and thus enabling acute sexual discrimination in males living in a group with females.

**Figure 6:**
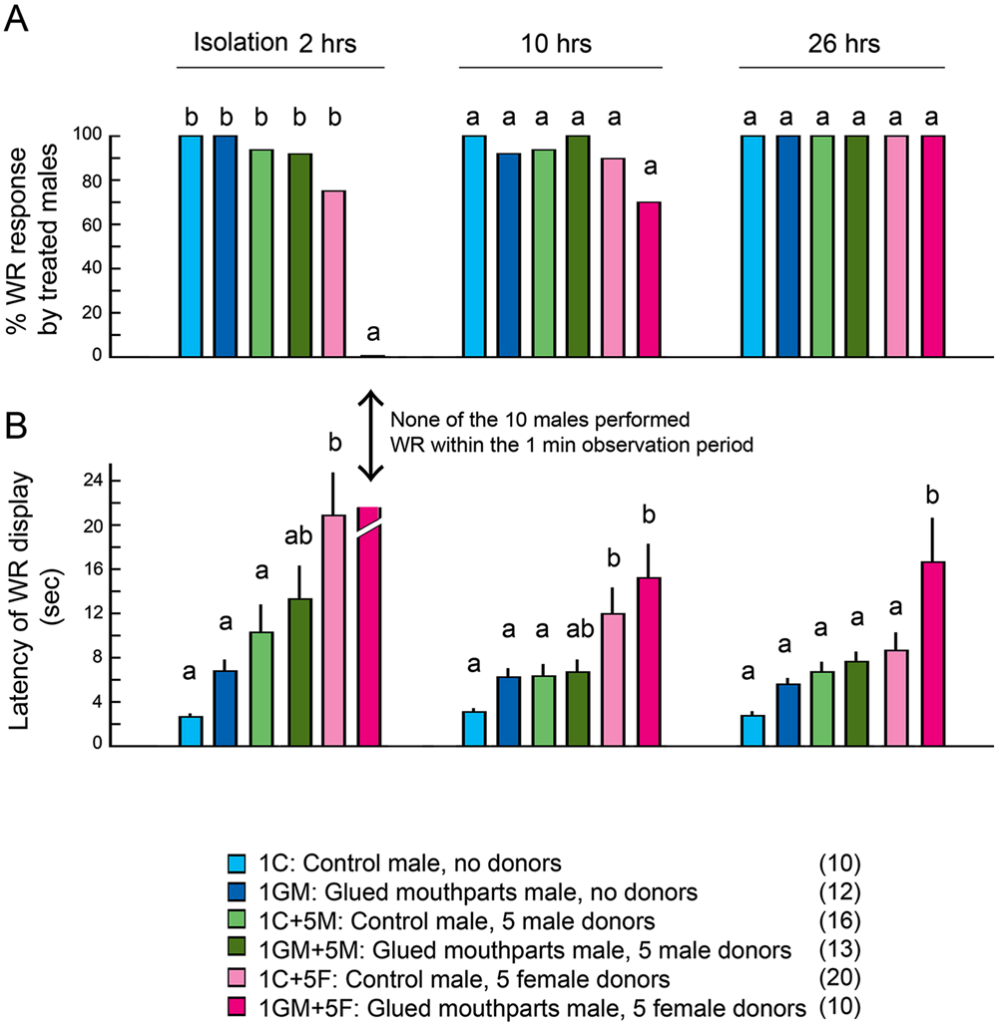
Courtship performance of treated males in response to an intact female antenna. (**A**) Percentage of treated males that responded with a WR display. Males that were prevented from grooming (1GM + 5 F) were contaminated with female pheromone and failed to display WR responses to a female antenna within 2 hrs of being separated from the female group. All treated males recovered after 26 hrs of isolation. (**B**) Latency of the WR response (mean ± SE). The freely grooming males contaminated with female cuticular lipids (1 C + 5 F) had significantly longer WR latencies after 2 and 10 hrs of isolation, but recovered after 26 hrs of isolation. The latency to WR of similarly treated males, but with glued mouthparts (1GM + 5 F), remained significantly higher than in control males even after 26 hrs of isolation. Different letters above the bars indicate significant differences (ANOVA with Tukey’s HSD, *p* < 0.05). Numbers in parentheses indicate number of insects tested. Results of the statistical analysis are shown in SI Table 4.

Figure 6 shows the WR responses of female-contaminated males to isolated female antennae. None of the males that were kept with females for 24 hrs while prevented from grooming (1GM + 5 F), and then isolated for 2 hrs, showed WR responses (Fig. 6A and SI Table 4). The isolated female antenna elicited WR from >70% of the males in the other treatment groups. This indicates that group-living with females caused a large decline in male courtship response when males could not groom their antennae. The WR response recovered to 100% in all treatment groups after 26 hrs of isolation. Thus, while grooming is critical to maintain antennal sensitivity and a high courtship response, isolation from females facilitates a recovery of the sexual response even when the antenna cannot be groomed. These results suggest that either other mechanisms, such as enzymatic degradation, clear the female pheromone from the male antenna, or the male’s CNS becomes more responsive during isolation.

Wing-raising latency was a more sensitive measure of the effects of these treatments. Although the WR response fully recovered after 10 hrs of isolation in mouthparts-glued males that were kept with females for 24 hrs (1GM + 5 F) (Fig. 6A and SI Table 4), the latency of their sexual response remained significantly higher after 10 and 26 hrs of isolation (Fig. 6A and SI Table 4). Even control males that could groom their antennae had significantly longer WR latency 2 hrs after being housed with females for 24 hrs (1 C + 5 F) than did isolated males (1 C) or males housed with five males (1 C + 5 M) for 24 hrs. These results indicate that although isolation from females helps males recover their courtship response, antenna grooming enables a faster recovery of a quicker courtship response, which is beneficial in male-male competition.

### Contamination of male antennae with female pheromone may lead to CNS habituation and peripheral sensory adaptation

**Figure 7:**
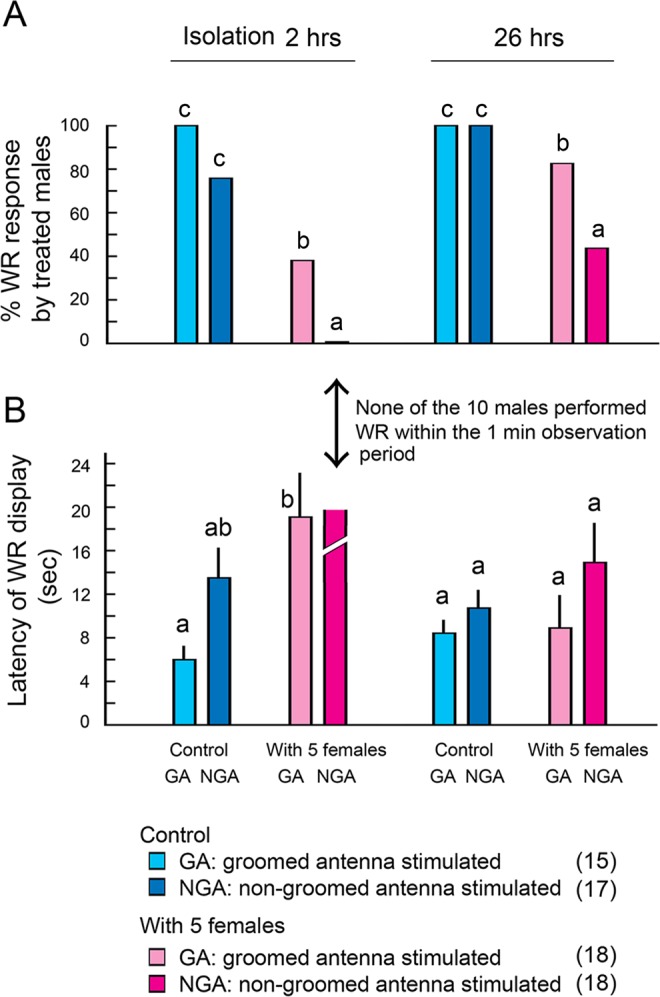
Courtship performance of males with one groomed and one non-groomed antenna in response to an intact female antenna. (**A**) Percentage of treated males that responded with a WR display. (**B**) Latency of the WR response (mean ± SE). The non-groomed antenna of males exposed to 5 females for 24 hrs was unresponsive to an intact female antenna even after the male was isolated from the females for 2 hrs. The groomed antenna of similarly treated males was also less responsive than the groomed antenna of males that were housed alone. Thus, both CNS habituation and peripheral adaptation appear to be responsible for lower courtship performance when males aggregate with females. Antenna grooming mitigates these effects and is a critical adaptation for males to maintain courtship performance while aggregating with females. After 26 hrs of isolation, all males in all treatments recovered their courtship responses, showing that isolation from females results in heightened courtship performance. Different letters above the bars indicate significant differences (ANOVA with Tukey’s HSD, *p* < 0.05). Numbers in parentheses indicate number of insects tested. Results of statistical analysis are shown in SI Table 5.

We hypothesized that residual female pheromone on non-groomed male antennae continuously stimulated the antennal chemosensory system, affecting courtship responses through two mechanisms: peripheral sensory adaptation of sex pheromone-sensitive antennal neurons and habituation of CNS perception of the female sex pheromones. Direct experimentation by recording neural responses could not be done because contact sex pheromone sensitive neurons have not been identified in *B*. *germanica*. Instead we developed an assay paradigm using single males with one antenna that was prevented from grooming and one freely groomed antenna. The results revealed that >70% of the control males that were housed alone responded with WR when either their groomed antenna (GA, 100% response) or non-groomed antenna (NGA, 76.5% response) was stimulated with a female antenna (Fig. 7A and SI Table 5). On the other hand, males that were housed for 24 hrs with five females had significantly lower WR performance after 2 hrs of isolation, with only 38.9% responding when their groomed antenna was stimulated and none responded when their non-groomed antenna was stimulated. Stimulation of the female-exposed groomed antenna 2 hrs after isolation from females elicited significantly lower WR than stimulation of groomed antennae that were free of female pheromone. These patterns were also reflected in WR latency, with the groomed antenna of female-exposed males eliciting WR with longer latency than the groomed antenna of isolated males (Fig. 7B and SI Table 5). The lack of response from the non-groomed antenna of female-exposed males, and significantly higher WR responses elicited by the contralateral groomed antenna, suggest that differential adaptation of female sex pheromone sensing neurons may be responsible. The diminished WR responses elicited by the groomed antenna suggested that CNS habituation to the female contact sex pheromone was also involved.

After 26 hrs of isolation, the WR performance of all males improved (Fig. 7 and SI Table 5), indicating that isolation from females facilitated the recovery of courtship performance. However, the importance of antennal grooming was highlighted by the slow recovery of courtship performance of grooming-incapable antennae even after 26 hrs of isolation from females.

## Discussion

Cuticular lipids, including cuticular hydrocarbons (CHCs), often function as sex pheromones in insect chemical communication^1^. In this study, we used the German cockroach to test whether males contaminated by female cuticular lipids in aggregations experience a decline in courtship performance, and whether grooming the antennae effectively removes the contaminants from the male’s antennae, maintains chemosensory acuity, and preserves courtship performance.

### Both antenna grooming and mating are more frequent in the scotophase

Grooming behavior effectively removes environmental physical contamination from animals^16–21^. In the American cockroach, *Periplaneta americana*, antenna grooming also effectively removes excess cuticular lipids from the antennae and thus enhances their olfactory acuity for a range of behaviorally relevant chemicals, including the sex pheromone^22,23^. For antenna grooming to be most effective, this behavior should be expressed most when olfactory-guided behaviors are expressed. We used male German cockroaches to test the hypothesis that an elevated frequency of antennal grooming should coincide with greater sexual activity in this nocturnal insect. We found that both grooming behavior, especially antenna grooming, and mating activity increased in the scotophase. Moreover, using antennae that could not be groomed, we showed that the accumulation of CHCs on the antennae was greater in scotophase than in photophase. These results suggest that two related processes are particularly active in the scotophase to enhance the sensitivity and selectivity of the chemosensory system to guide behaviors such as mate-recognition: males (a) deposit fresh cuticular lipids on the antenna, and (b) actively groom the antennae to keep their chemosensory system clean.

### Antenna grooming in males removes contaminating female cuticular lipids

When grooming was prevented by sealing the mouthparts, males were prevented from removing excess CHCs that accumulated on their antennae. These males also could not remove other chemical contaminants, including female sex pheromones, from their non-groomed antenna, as shown in Fig. 5. Thus, without grooming each male antenna accumulated 0.9 ng of female pheromone, 30-fold more than the 0.027 ng in control males that could groom their antennae. Notably, whereas each female antenna contains 10.9 ng of 3,11-dimethylnonacosan-2-one, the effective dose that stimulates 50% of males to court (EC_50_) is 1.1 ng^24^. It is not surprising, therefore, that these female-contaminated males could elicit courtship responses from normal males.

The behavioral effects of the inability to groom antennae were best illustrated with experimental males with one antenna that could be freely groomed and a contralateral antenna that was prevented from being groomed. When the male was housed with five females for 24 hrs, his non-groomed antenna became contaminated with female pheromone, and it elicited courtship from other males when used as an isolated stimulus in the ‘antenna-on-a-stick’ paradigm. The contralateral male antenna that was freely groomed elicited a courtship response from only one of 20 males, with long latency to wing-raising. This result underscores the effectiveness of grooming in removing chemical contaminants from the antennae, which enables efficient chemical communication, including sexual recognition and discrimination, within complex mixed sex and stage aggregations.

In a recent study using the American cockroach, we demonstrated that olfactory neurons of non-groomed antennae had lower electrophysiological responses than olfactory neurons of freely groomed antennae to both general odors and to a volatile sex pheromone^22^. This was attributed to accumulation of excess CHCs that covered olfactory sensilla on the antenna and functioned as a physical barrier to prevent odor molecules from reaching the olfactory neurons. In that study, however, we did not relate the neural impairment to any behavioral deficiency. Here, we found that male antennae that were prevented from grooming and then naturally contaminated with female pheromone in an aggregation lost the ability to drive male courtship even after 2 hrs of isolation from females (Fig. 6). We suspected that at least two mechanisms contributed to this ‘anosmia’: (a) sensory and CNS changes due to prolonged presence of female pheromone on the antenna (further discussed below), and (b) accumulation of excess cuticular lipids on the non-groomed antenna interfered with detection of female contact sex pheromone. The latter is supported by our observation that courtship latency of isolated males that could not groom their antennae was slightly longer than in isolated males that freely groomed their antennae (Fig. 6). Additionally, even when males were housed with other males, with their antennae either freely groomed or prevented from being groomed, their courtship latencies were longer than the respective control males that were continuously isolated without contact with other males. We suspect that during interactions with other males, the antennae of the test (recipient) male received excess cuticular lipids from the other males, and this contributed a physical barrier that decreased the acuity of chemosensory detection.

Prolonged presence of residual female pheromone on male antennae continuously stimulates the male’s sex pheromone sensitive neurons and diminishes courtship performance through changes in peripheral sensory responses and/or CNS responses. The results of bioassays using intact males with one freely groomed antenna and a contralateral non-groomed antenna (Fig. 7) suggest that both sensory adaptation and CNS habituation result in diminished behavioral responses. These observations indicate that the function of grooming is to enhance sensory acuity not only by removing excess endogenous cuticular lipids that can deny odorants access to sensory neurons, but also by removing chemical contaminants to avoid sensory adaptation and CNS habituation to behaviorally important odorants, including semiochemicals. The latter function is aided by enzymatic deactivation and clearance of odorants within the sensillar lymph^15^ and possibly on the cuticular surface^25^.

In addition to biochemical and grooming-related upkeep of the antennae, isolation from females was effective at expediting the recovery of courtship performance. Full recovery after 24 hrs with females was observed after 10 hrs of isolation in males both with and without grooming (Figs 6 and 7). One possibility is that even without antennal grooming the continuous accumulation of endogenous cuticular lipids slowly removed the sex pheromone contamination from the male antennae. A second possibility is that during isolation the males physically contacted the cotton plug and the clean glass walls of the test tube, which adsorbed and thus reduced both female pheromone and male cuticular lipids on the male’s antennae. Finally, it is possible that the sensitivity and selectivity of the chemosensory system to contact sex pheromone is modulated during isolation, possibly through differential expression of chemosensory-perception genes, as in other insects, e.g.^26^. The mechanisms that facilitate the recovery of the courtship response without antennal grooming should be explored in future work; however, our results indicate that antenna grooming plays a critical function in the rapid recovery of courtship performance.

## Conclusions

In this study we demonstrated the impact of antennal contamination and the function of antenna grooming using the female contact sex pheromone of the German cockroach and male courtship behavior as a model system. In natural situations, a vast array of environmental chemicals can contaminate the chemosensory system during routine animal activity, such as foraging, interactions with predators and prey, manipulation of objects in the environment (soil, leaves), and social interactions with conspecifics. Chemical residues, and particularly semiochemicals, may accumulate on chemosensory appendages and cause a decline in performance of chemosensory-guided behaviors because of sensory adaptation and CNS habituation. Secretion of new CHCs on the chemosensory structures can serve to refresh the cuticular lipids, but concomitant physical grooming is required to remove foreign chemicals and enable a turnover of cuticular lipids. Together, the secretion of new cuticular lipids, which may also contain factors that aid in degradation of foreign chemicals, and physical grooming, maintain the acuity, sensitivity and selectivity of the chemosensory system. The mechanisms that maintain the cuticular lipid layer are especially important in group-living and social insects, where semiochemicals that convey information about sex, stage, kin, nestmate and fertility status need to be clearly detected, recognized and drive specific and appropriate behaviors with minimal interference and ambiguity. Since their body surface is readily contaminated with conspecific cuticular lipids via physical interaction, gregarious insects, such as the German cockroach, that do not engage in allogrooming, must maintain their cuticular lipids by self-grooming to increase the chemical signal/noise ratio among group members and maintain individual-based chemical communication. On the other hand, in eusocial insects such as ants, that detect differences between CHC profiles using the antennal chemosensory system to discriminate non-nestmates from nestmates^5,27^, workers in the same colony exchange their individual CHCs and pool the blended CHCs in the postpharyngeal gland^28,29^. Then they distribute and share the CHC blends as a ‘nestmate uniform’ on all nestmates^27,28^. Self-grooming, allogrooming, trophallaxis and secretion of fresh CHCs in ants serve to maintain a homogeneous cuticular lipid layer^28,29^ and may contribute to increasing the chemical signal/noise ratio among different colonies for maintaining colony-based chemical communication.

## Methods

### Insects

A laboratory strain of *Blattela germanica* (American Cyanamid, collected in a Florida apartment >70 yrs ago) was reared on water and dry food pellets (Purina No. 5001 Rodent Diet, PMI Nutrition International, St. Louis, MO) in a walk-in chamber with infrared lights, 27 ± 1 °C, 40–70% relative humidity, and L:D = 12:12 photoperiod (Light, 20:00–08:00). Newly emerged adult males and females were kept separately to prevent contact. Sexually mature virgin males (10–12 days old) were used in all experiments. Sexually immature and unreceptive virgin females (0 day old) were used in time-course observations of courtship and mating success. Sexually mature and receptive virgin females (5–6 days old) were used in all other experiments. All behavioral assays were carried out during the scotophase in the walk-in chamber using an IR-sensitive camera (Everfocus EQ. 610 Polestar, Taiwan).

### Observations

#### Experiment 1: Observations of mating in relation to photocycle

To determine when in the photocycle males are motivated to court females and when females accept males, pairs of 65 sexually mature virgin males and 65 sexually immature virgin females were video-recorded for 7–10 days (Fig. 1B). Pairs were kept in petri dishes (100 × 15 mm) with food and water. The number of pairs that mated was compared between early photophase (20:00–02:00), late photopase (02:00–08:00), early scotophase (08:00–14:00) and late scotophase (14:00–20:00). The results were compared by Chi-square test (α = 0.05).

#### Experiment 2: Observations of locomotor activity and grooming behavior

A single male was placed in a clean hexane-washed glass test tube (2.5 cm diameter × 15 cm long) with a clean cotton plug (Fig. 1C and SI Fig. 1A). The test tubes were placed horizontally, and test males were kept in the tubes without food and water for 24 hrs. Twelve individual 10–12 days old males were continuously video-recorded, but data were analyzed from representative 1 hr segments at four different time points throughout the photocycle: Experimental Day0: 18:00–19:00 (late scotophase), Day0: 22:00–23:00 (early photophase), Day0: 06:00–07:00 (late photophase), and Day1: 10:00–11:00 (early scotophase). The proportion of locomotor activity was obtained from each tested male as (Total observation time [60 min] – Total resting time)/Total observation time [60 min]. Resting was defined as no movement of all body parts. After applying angular transformation to the proportion, results were compared among the four different time points by ANOVA, Tukey’s HSD (α = 0.05). Grooming frequencies of the antennae, forelegs, midlegs, hindlegs and abdomen including cerci were compared using ANOVA, Tukey’s HSD (α = 0.05) (Fig. 1C bottom). Since cockroaches groom different body parts in different manners, we did not compare the time spent grooming different body parts. Also, since the grooming frequencies for midlegs and hindlegs were low, these data were combined. Based on these results, we decided to carry out all bioassays for WR in the scotophase.

### Preventing grooming and quantification of cuticular hydrocarbons (CHCs)

#### Experiment 3: Designing an effective method to prevent grooming

First, we tested which body parts are important for grooming to design an effective method to prevent grooming (Fig. 2) and SI Fig. 1B). After 24 hrs starvation (experimental Day0: 08:00 to Day1: 08:00), 10–12 days old male cockroaches were placed into five groups: a freely grooming group (control, n = 5) and four experimentally manipulated groups to prevent grooming (n = 5 for each treatment group). Males in all five groups were briefly anesthetized with carbon dioxide and kept on the ice. In three of four treatment groups, a pair of body parts was ablated with fine scissor: forelegs ablated at the coxae (FC, group 1); forelegs ablated at the trochanter (FT, group 2); forelegs ablated at the trochanter and maxillary palps ablated (FTM, group 3). Additionally, in one treatment group, the males’ mouthparts were glued with cyanoacrylate gel (Loctite super glue, Henkel, Ireland) (GM, group 4). These treatments did not affect locomotor activity or survivorship over the short course of our experiments. Each male from the control and treatment groups was placed individually in a glass test tube stoppered with a cotton plug at experimental Day1: 08:00. The tubes were placed horizontally. The insects moved freely in the test tube during the 24 hr period (Day1: 08:00 to Day2: 08:00).

Since prevention of grooming results in accumulation of CHCs on the cuticular surface^22^, we measured the total amount of CHCs on each male in the five treatment groups and considered that the treatment resulting in the highest accumulation of CHCs was most effective in preventing grooming. Males were quickly killed at −80 °C, thawed to room temperature, and carefully dissected, separating various body parts with hexane-washed forceps. Body parts were extracted and CHCs quantified by gas chromatography (GC, below), and the amounts of CHCs were compared among treatments (ANOVA, Tukey’s HSD, α = 0.05) (Fig. 2). The results indicated that gluing the mouthparts resulted in the largest accumulation of CHCs, so we used this method to prevent grooming in all bioassays, except in Experiments 5 and 9.

#### Experiment 4: CHC accumulation in scotophase and photophase

To test for a correlation between grooming activity and CHC accumulation, we compared the CHCs of freely grooming (control) and grooming-prevented (glued mouthparts) males in the scotophase and photophase (Fig. 3A and SI Fig. 1C); CHCs were extracted from the antennae and the rest of the body. After 24 hrs of starvation, the control and treated groups were prepared as described in Experiment 3. Insects were left in test tubes for 12 hrs either in the scotophase (Day1: 08:00 to Day1: 20:00; 10 control males, 9 treated males) or photophase (Day1: 20:00 to Day2: 08:00; 10 control males, 10 treated males). Insects were quickly killed at −80 °C and the antennae were carefully separated from the body. The amounts of CHCs were compared by ANOVA, Tukey’s HSD (α = 0.05) (Fig. 3A).

#### Experiment 5: CHC accumulation on various body parts

To test if chemosensory appendages accumulate relatively more CHCs than other body parts, the amounts of CHCs on ten body parts (maxillary palps, antennae, head without maxillary palps and antennae, forelegs, midlegs, hindlegs, forewings, hindwings, cerci, and the rest of the body) were analyzed using control and treatment groups (Fig. 3B and SI Fig. 1B). Grooming was prevented by two methods in non-groomed groups: using 13 males, each male with glued mouthparts was immobilized in a pipette tip with the head exposed outside of the pipet tip; using 27 males, the male was placed in the pipet tip with just the cerci exposed outside the pipet tip. Insects in pipet tips could not move and groom and were prevented from removing CHCs by contact with the glass tube and cotton plug used in previous experiments. For groomed insects, two control groups were prepared using 10 males for comparison with the first treatment group (head out of pipet tip) and 20 males for comparison with the second treatment group (cerci out of pipet tip). Control insects were kept individually in test tubes but otherwise experienced the same conditions as the treatment groups. All insects were kept for 24 hrs (Day1: 08:00 to Day2: 08:00), quickly killed at −80 °C, and different body parts were dissected for CHC analysis. The amounts of CHCs on different body parts were compared between the treatment groups and respective control (unpaired Student’s *t*-test, α = 0.05) (Fig. 3B).

### Gas chromatographic analysis of CHCs and female contact sex pheromone

#### For Experiments 1–5

Small body parts, such as antennae, maxillary palps, labial palps, head, legs, wings, and cerci, were extracted for 2 min in 0.1 ml hexane containing an internal standard (100 ng *n*-hexacosane [*n*-C26]). The rest of the body was extracted for 2 min in 1 ml hexane (10 µg *n*-C26 internal standard). Extracts were analyzed on a DB-5 column (20 m × 0.18 mm internal diameter ×0.18 µm film thickness; J&W Scientific) in an Agilent 7890A gas chromatograph equipped with a flame ionization detector (GC-FID) with a 7683B Agilent auto sampler controlled by Chemstation (Agilent Technologies). Ultrahigh-purity hydrogen was used as carrier gas (0.75 mL/min constant flow rate). The inlet was held at 300 °C, FID at 320 °C, and oven temperature was 80 °C (1 min)–10 °C/min–310 °C (20 min hold). Total peak area was used for the calculation of total CHC amount. The specific profiles of CHCs on different body parts were not considered in these experiments.

#### For Experiment 7

Antennae were ablated from individual cockroaches and 5 pairs of antennae were pooled for each of 5 replicates. Antennae were extracted in conical glass vials for 2 min in 200 µL of hexane containing 100 ng of *n*-C26 and 10 ng of heptacosan-14-one (C27–14-one) as internal standards. The extracts were then fractionated by flash chromatography: disposable borosilicate glass Pasteur pipettes with 200 mg of chromatographic silica gel (100–200 mesh, Fisher Scientific, NJ, U.S.A.) were activated at 110 °C and washed with 2 ml hexane (Optima, Fisher Scientific). The extract was then loaded onto the column and eluted with 2 ml hexane to recover the CHCs, then 2 ml of 10% diethyl ether (Fisher Scientific) in hexane to recover the methyl ketone contact sex pheromone. The two fractions were reduced to dryness with a gentle stream of high purity nitrogen, reconstituted in octane, and transferred to a 50 µL glass insert in an autoinjection 1.5 ml vial. For CHC analysis we injected 3 of 10 μL, and for pheromone analysis 3 of 3 μL were injected in splitless mode using the procedures detailed for Experiments 1–5. The column was held at 50 °C for 1 min, increased to 320 °C at 10 °C/min, and held at 320 °C for 10 min. For Principal Components Analysis (PCA), we used the percentage of each of 29 peaks previously identified by Jurenka *et al*.^30^. PCA was conducted in JMP (JMP Pro 13.1.0, SAS Institute, Inc., Cary, NC). The amount (mass) of each peak was determined relative to the internal standards. Differences among treatments were analyzed by ANOVA and Tukey’s-Kramer Honestly Significant Difference test.

### Bioassays

#### Experiment 6: Context of the wing-raising display

To establish that the WR display occurs only when a male is stimulated with a female antenna (SI Video 1), we assayed males and female using an isolated antenna from a sexually mature virgin male or female (Fig. 4 and SI Fig. 1D). Each test insect of 20 males and 20 females was kept in a glass test tube without food and water for 24 hrs, as in Experiment 2. A stimulus antenna was prepared by freshly ablating an antenna from either a male (10–12 days old) or female (5–6 days old) and attaching it to the tip of a glass Pasteur pipette with wax (Soft Plate Wax, GC Corp., Tokyo, Japan). This freshly prepared ‘antenna-on-a-stick’^10^ was used in scotophase to contact the antennae of individual test insects in test tubes to stimulate the WR display. The observation period was 1 min after the start of stimulation, and observations stopped when the test insect displayed WR. A single stimulus antenna was tested on a single test insect, and neither stimulus antenna nor tested insects were reused. The WR performance was evaluated using two parameters: The proportion of insects that responded to the stimulus antenna was tested by Chi-square test (α = 0.05) (Fig. 4). The latency (sec) of the WR display was obtained from the start of stimulation to the initiation of the display.

#### Experiment 7: Impact of contamination with female cuticular lipids on sexual discrimination

We carried out bioassays to test whether males in a group of females are contaminated with female cuticular lipids, and whether such contamination disrupts the courtship of other males in the group (Fig. 5 and SI Fig. 1E). Recipient males were housed alone or with either five female- or five male-donors that could transfer their cuticular lipids to the recipient. In all assays, males were 10–12 days old and females 5–6 days old. The procedures were as follows: Donors were starved for 24 hrs (experimental Day0: 08:00 to Day1: 08:00). Recipient males were separated into six treatment groups and maintained individually in test tubes for 24 hrs (Day1: 08:00 to Day2: 08:00). The six treatment groups of recipient males included: single freely groomed control (1 C); single grooming-prevented mouthparts glued male (1GM); a single C recipient male kept with five donor males (1 C + 5 M); a single GM male kept with five donor males (1GM + 5 M); a single C recipient kept with five donor females (1 C + 5 F); and a single GM recipient male kept with five donor females (1GM + 5 F). The recipient male in each treatment was placed in the test tube as described in Experiment 2. To discriminate the recipient male from donor males, the tips of the wings of the donor males were cut. To prevent mating of the recipient male and donor females, the genitalia of donor females were blocked with super glue. Recipient males were exposed to the six treatments for 24 hrs. Concurrently, 20 test males (10–12 day-old) were acclimated individually in test tubes for 24 hrs (experiment time Day1: 08:00 to Day2: 08:00), as in Experiment 2, during which time they could freely groom. After 24 hrs, the antennae of the recipient males were freshly ablated and used as WR stimuli of intact test males. Bioassays of WR with these test males started in the early scotophase (08:00) of experimental Day2. The stimulation procedure was the same as in Experiment 6. The proportion of responders showing WR display was tested by Chi-square test (α = 0.05) (Fig. 5). The WR latency (sec) was defined from the start of stimulation to the initiation of WR. Normally, males do not show WR to the antennae of sexually mature males, as shown in Experiment 6. Therefore, we considered that when the isolated antenna of a recipient male elicits WR from test males, the recipient male’s antennae had been contaminated with female pheromone. Additionally, we measured the amounts of 3,11-dimethylnonacosan-2-one (methyl ketone, a major component of the female contact sex pheromone) and total CHCs per antenna of males and females. Five pairs of antennae of males in four treatment groups (1 C, 1GM, 1 C + 5 F and 1GM + 5 F) and females (5 F, five freely groomed females without males in a test tube for 24 hrs) were prepared and were analyzed by gas chromatographic analysis.

#### Experiment 8: Impact on males of being contaminated with female pheromone)

In Experiment 7 we demonstrated that males prevented from grooming could not remove female pheromone contamination from their antennae (Fig. 6 and SI FIg. 1F). Next, we tested the impact of female pheromone contamination on the courtship performance of contaminated males. We prepared the recipients and donors using the same procedures as in Experiment 7, but the contaminated recipient was then used as the test male for WR bioassays. After 24 hrs of co-habitation with either males or females, each test male (recipient) was isolated in a new test tube, at 08:00 of experimental Day2. The test male was then stimulated after 2, 10 and 26 hrs (Day2: 10:00, 18:00 and Day3: 10:00) with an antenna obtained from a 6 day old virgin female (that is expected to elicit WR in nearly 100% of sexually mature males). At least 15 test males were used in each treatment group. The proportion of males that displayed WR was tested by Chi-square test (α = 0.05) (Fig. 6A). The WR display latency (sec) was tested by unpaired Student’s *t*-test (α = 0.05) and ANOVA (α = 0.05. Tukey’s HSD) (Fig. 6B and SI Table 4).

#### Experiment 9: Mechanisms for the decline in courtship performance in female-contaminated males

In Experiment 8, males that were prevented from grooming were contaminated with female cuticular lipids, which greatly diminished their courtship response (Fig. 7 and SI Fig. 1G). We hypothesized that the decline in WR performance was mediated by the residual female pheromone on the male’s antennae, either through peripheral sensory adaptation or CNS habituation, or both. We designed an experiment that could test for adaptation of one antenna but not the other, in the same male. A small ring of plastic pipette tip was attached to the base of either the right or left antenna with super glue and it served as an antennal collar. The treated male could not groom the treated antenna, because the collar at its base prevented the antenna from reaching the mouth; the untreated antenna was free to be groomed and thus served as control. We predicted that after co-habitation with females and contamination with female pheromone, the treated (grooming-prevented) antenna would remain contaminated whereas the contralateral antenna would be groomed and freed of female pheromone. Thus, stimulation of each of the two antennae would elicit differential WR performance if antennal sensory adaptation were involved. On the other hand, if the residual female pheromone on the non-groomed antenna caused CNS habituation, stimulation of either antenna should fail to elicit WR. These males, with one immobilized antenna, were separated into two groups: In the first group, a single male was housed alone in a test tube (Control) with either his non-groomed antenna stimulated (NGA, 17 males: 10 with left antenna glued and 7 with right antenna glued) or his groomed antenna stimulated (GA, 15 males: 6 with left antenna glued and 9 with right antenna glued). In the second group, a single male was housed in a test tube with five females for 24 hrs, and then either his non-groomed antenna was stimulated (NGA, 18 males: 9 with left antenna glued and 9 with right antenna glued) or his groomed antenna stimulated (GA, 18 males: 9 with left antenna glued and 9 with right antenna glued). The test males were prepared using the same procedure as in Experiment 7. After 24 hrs alone or with five females, each test male was isolated in a new test tube at 08:00 of experimental Day2. Bioassays were conducted 2 hrs (Day2: 10:00) and 26 hrs (Day3: 10:00) later in plastic arenas (10 × 10 × 7.5 cm) and either the non-groomed or groomed antenna was stimulated with an antenna from a 6 day old virgin female, using the procedures outlined in Experiment 6. The proportion of males that responded with a WR display was tested by Chi-square test (α = 0.05) (Fig. 7A). The WR latency (sec) was tested by unpaired Student’s *t*-test (α = 0.05) (Fig. 7B).

## Supplementary information

SI Video1 Context of the wing-raising display of male German cockroach

ESM containing SI Tables and Figure

Dataset 1

## Data Availability

The data supporting the findings in this study are available as Supplementary Information.
