# Supplementary material for: Antennal grooming facilitates courtship performance in a group-living insect, the German cockroach Blattella germanica
Source: Sci Rep. 2019 Feb 27;9:2942. doi: 10.1038/s41598-019-39868-x (PMC6393502; doi:10.1038/s41598-019-39868-x)
Supplement: Supplementary file 2 — ESM containing SI Tables and Figure [file 41598_2019_39868_MOESM2_ESM.pdf]

Electric supplementary material (ESM)

Antennal grooming facilitates courtship performance in a group-living insect, the German cockroach *Blattella germanica*

Ayako Wada-Katsumata,[1] & Coby Schal[1]

[1] North Carolina State University, Department of Entomology and Plant Pathology and W.M. Keck Center for Behavioral Biology, Raleigh, North Carolina, USA

Correspondence and requests for materials should be addressed to A.W-K. (email: akatsum@ncsu.edu) or C.S. (email: coby@ncsu.edu)

SI Table 1 Statistical analysis results for Fig. 3B

Factor: CHC amount (non-groomed vs groomed)

Treatment	Statistical test	df	T value	*p*
Antenna	Unpaired Student's *t*-test	21	-12.719	< 0.01
Maxillary palps	Unpaired Student's *t*-test	21	-10.16	< 0.01
Head	Unpaired Student's *t*-test	21	-6.5125	< 0.01
Forelegs	Unpaired Student's *t*-test	21	-12.265	< 0.01
Midlegs	Unpaired Student's *t*-test	21	-12.386	< 0.01
Hindlegs	Unpaired Student's *t*-test	21	-10.118	< 0.01
Cerci	Unpaired Student's *t*-test	45	-3.2443	< 0.01
Forewings	Unpaired Student's *t*-test	21	-4.7125	< 0.01
Hindwings	Unpaired Student's *t*-test	21	-1.1492	0.2634
Rest of body	Unpaired Student's *t*-test	21	-6.1149	< 0.01

SI Table 2 Total CHC and 3,11-dimethylnonacosan-2-one (methyl ketone, a major component of the female sex pheromone) per antenna and statistical analysis for Fig. 5A

1C = control male
F = control female
1C+5F = control male with 5 females
1GM = male with glued mouthparts
1GM+5F = male with glued mouthparts with 5 females

Treatment	Methyl ketone (ng / antenna)	SEM	CHCs (µg / antenna)	SEM
1C	0.027	0.010	0.909	0.018
1GM	0.151	0.033	2.540	0.060
1C+5F	0.065	0.029	0.752	0.009
1GM+5F	0.901	0.116	2.260	0.143
5F	10.888	0.760	1.017	0.033

Statistical analysis for methyl ketone

Factor	Statistical test	df	F value	*p*	Post hoc test	Pairwise comparison	*p*
Methyl ketone	ANOVA	3	43.6008	< 0.0001	Tukey's HSD	1C vs 1GM	0.5283
						1C vs 1C+5F	0.9724
						1C vs 1GM+5F	< 0.0001
						1GM vs 1C+5F	0.7784
						1GM vs 1GM+5F	< 0.0001
						1C+5F vs 1GM+5F	< 0.0001

SI Table 3 Percentage representation of the antennal CHC amount for Fig. 5

Each GC peak is represented as a percentage of the total of 29 hydrocarbons. The peak numbers correspond to the CHCs identified by Jurenka et al. (1989). Peak 15 (9-, 11-, 13-, and 15-methylnonacosane) is known as a male-enriched CHC, and peak 22 (3,7-, 3,9-, and 3,11-dimethylnonacosane) is a female-enriched CHC. 3,11-Dimethylnonacosane also serves as precursor to several components of the female contact sex pheromone.

	Treatment group									
**Hydrocarbon**	**1C**	**SEM**	**5F**	**SEM**	**1C+5F**	**SEM**	**1GM**	**SEM**	**1GM+5F**	**SEM**
1. n-Heptacosane	1.00	0.03	0.87	0.04	0.94	0.06	0.77	0.06	0.76	0.01
2. 11- and 13-Methylheptacosane	3.13	0.07	1.50	0.18	3.58	0.18	2.31	0.09	2.28	0.06
3. 5-Methylheptacosane	1.85	0.05	1.24	0.14	2.04	0.10	1.46	0.03	1.49	0.03
4. 11,15-Dimethylheptacosane	0.47	0.01	0.42	0.03	0.52	0.03	0.39	0.01	0.41	0.01
5. 3-Methylheptacosane	3.37	0.04	2.36	0.11	3.28	0.15	2.95	0.08	2.82	0.06
6. 5,9- and 5,11-Dimethylheptacosane	1.64	0.04	2.27	0.27	1.98	0.07	1.39	0.04	1.56	0.02
7. n-Octacosane	0.73	0.02	0.74	0.04	0.60	0.03	0.60	0.03	0.60	0.01
8. 3,11- and 3,9-Dimethylheptacosane	1.71	0.04	2.09	0.12	1.91	0.10	1.51	0.04	1.65	0.04
9. 12- and 14-Methyloctacosane	1.30	0.01	0.98	0.06	1.36	0.02	1.24	0.02	1.23	0.01
10. 2-Methyloctacosane	0.90	0.01	1.06	0.02	0.87	0.01	0.83	0.01	0.87	0.01
11. 4-Methyloctacosane	0.75	0.00	0.83	0.02	0.71	0.01	0.73	0.01	0.73	0.00
12. Unknown	0.27	0.01	0.39	0.02	0.28	0.01	0.26	0.01	0.29	0.00
13. n-Nonacosane	5.75	0.20	7.79	0.56	5.12	0.21	4.92	0.17	5.29	0.25
14. Unknown	0.45	0.01	0.55	0.02	0.49	0.01	0.48	0.01	0.50	0.01
15. 9-, 11-, 13-, and 15-Methylnonacosane	22.11	0.27	14.73	0.50	23.03	0.37	24.70	0.26	22.93	0.16
16. 7-Methylnonacosane	4.01	0.11	3.40	0.07	4.03	0.06	4.11	0.04	3.88	0.10
17. 5-Methylnonacosane	7.41	0.08	7.02	0.26	7.19	0.19	7.48	0.17	7.26	0.04
18. 13,17- and 11,15-Dimethylnonacosane	4.99	0.06	6.87	0.28	5.47	0.12	4.96	0.05	5.39	0.07
19. Unknown (not used)	.	.	.	.	.	.	.	.	.	.
20. 3-Methylnonacosane	13.62	0.11	15.16	1.07	12.19	0.20	12.79	0.16	13.01	0.28
21. 5,9- and 5,11-Dimethylnonacosane	3.43	0.04	4.54	0.17	3.61	0.06	3.48	0.07	3.65	0.09
22. 3,7-, 3,9-, and 3,11-Dimethylnonacosane	15.45	0.16	18.81	0.24	15.29	0.23	16.04	0.21	16.96	0.17
23. Unknown	0.84	0.01	0.74	0.08	1.03	0.10	0.95	0.02	0.89	0.03
24. 11-, 13-, and 15-Methyltriacontane	1.27	0.03	1.82	0.06	0.77	0.29	1.31	0.02	1.46	0.02
25. Unknown	0.26	0.01	0.45	0.01	0.26	0.01	0.27	0.01	0.30	0.00
26. 4,8- and 4,10-Dimethyltriacontane	0.45	0.01	0.65	0.02	0.43	0.01	0.47	0.01	0.52	0.01
27. 11-, 13-, and 15-Dimethylhentriacontane	1.80	0.03	1.29	0.06	1.95	0.10	2.32	0.08	2.00	0.07
28. 13,17- and 11,15-Dimethylhentriacontane	0.36	0.01	0.53	0.03	0.41	0.01	0.44	0.01	0.46	0.01
29. 5,9- and 5,11-Dimethylhentriacontane	0.38	0.01	0.51	0.02	0.38	0.02	0.45	0.02	0.44	0.02
30. 10,12-Dimethyldotriacontane	0.29	0.01	0.40	0.01	0.30	0.01	0.36	0.01	0.36	0.02

Peak 19 was represented at <1% and inconsistently
1C = control male
F = control female
1C+5F = control male with 5 females
1GM = male with glued mouthparts
1GM+5F = male with glued mouthparts with 5 females
Colors correspond to chromatogram colors in Fig. 5A

SI Table 4 **Statistical analysis results for Fig. 6**

WR responses (Fig. 6A)
Factor: Effect of treatment on WR responses

Time after isolation	Statistical test	Df	χ^2 value	*p*
2 hrs	Chi-square	5	47.166	< 0.01
10 hrs	Chi-square	5		ns
26 hrs*				

*All tested insects responded
ns, not significant

Factor: Recovery of WR response (after 2 hrs, 10 hrs, 26 hrs of isolation)

Treatment	Statistical test	df	χ^2 value	*p*
1GM+5M	Chi-square	2	1.820	ns
1GM+5F	Chi-square	2	21.448	< 0.01
1GM	Chi-square	2	2.057	ns
1C+5M	Chi-square	2	0.915	ns
1C+5F	Chi-square	2	6.146	< 0.05
1C*				

*All tested insects responded
ns, not significant

WR display latency (Fig. 6B)
Factor: Effect of treatment on WR latency

Time after isolation	Statistical test	df	F value	*p*	Post hoc test	Pairwise comparison	*p*
2 hrs*	ANOVA	4	6.169	0.0003	Tukey's HSD	1C+5M vs 1C+5F	0.0378
						1C vs 1C+5F	0.0003
						1GM+5M vs 1C+5F	0.2819
						1GM vs 1C+5F	0.0047
						1C vs 1C+5M	0.3334
						1GM+5M vs 1C+5M	0.9399
						1GM vs 1C+5M	0.8941
						1GM+5M vs 1C	0.1058
						1GM vs 1C	0.8610
						1GM vs 1GM+5M	0.5141
10 hrs	ANOVA	5	5.162	0.0005	Tukey's HSD	1C+5M vs 1C+5F	0.1135
						1C vs 1C+5F	0.0068
						1GM+5F vs 1C+5F	0.8324
						1GM+5M vs 1C+5F	0.2182
						1GM vs 1C+5F	0.1633
						1C vs 1C+5M	0.7872
						1GM+5F vs 1C+5M	0.0280
						1GM+5M vs 1C+5M	1
						1GM vs 1C+5M	1
						1GM+5F vs 1C	0.0021
						1GM+5M vs 1C	0.7452
						1GM vs 1C	0.8535
						1GM+5M vs 1GM+5F	0.0527
						1GM vs 1GM+5F	0.0391
						1GM vs 1GM+5M	1
26 hrs	ANOVA	5	6.482	< 0.0001	Tukey's HSD	1C+5M vs 1C+5F	0.9413
						1C vs 1C+5F	0.1145
						1GM+5F vs 1C+5F	0.0097
						1GM+5M vs 1C+5F	0.9959
						1GM vs 1C+5F	0.7280
						1C vs 1C+5M	0.5705
						1GM+5F vs 1C+5M	0.0017
						1GM+5M vs 1C+5M	0.9995
						1GM vs 1C+5M	0.9965
						1GM+5F vs 1C	< 0.0001
						1GM+5M vs 1C	0.4358
						1GM vs 1C	0.8604
						1GM+5M vs 1GM+5F	0.0088
						1GM vs 1GM+5F	0.0006
						1GM vs 1GM+5M	0.9716

* 1GM+5F at 2 hrs: all tested insects did not respond

Factor: Recovery of WR latency (2 hrs, 10 hrs, 26 hrs after isolation)

Treatment	Statistical test	df	F value	*p*	Post hoc test	Pairwise comparison	*p*
1C	ANOVA	2	0.535	0.5920			
1GM	ANOVA	2	0.502	0.6100			
1C+5M	ANOVA	2	1.805	0.1770			
1GM+5M	ANOVA	2	3.209	0.0537			
1C+5F	ANOVA	2	5.84	0.0053	Tukey's HSD	2 hrs vs 10 hrs	0.0516
						2 hrs vs 26 hrs	0.0042
						26 hrs vs 10 hrs	0.6139
1GM+5F*	Unpaired Student's *t*-test 10 hrs vs 26 hrs	15	-0.2760	0.7862			

* 1GM+5F at 2 hrs: all insects did not respond

SI Table 5 Statistical analysis results for Fig. 7

WR responses (Fig. 7A)
Factor: Effect of treatment on WR responses

Time after isolation	Statistical test	df	χ^2 value	*p*
2 hrs	Chi-square	3	38.628	< 0.01
26 hrs	Chi-square	3	23.089	< 0.01

Factor: Recovery of WR responses (2 hrs, 26 hrs after isolation)

Treatment	Statistical test	df	χ^2 value	*p*
Control-GA*				
Control-NGA	Chi-square	1	2.550	ns
5Females-GA	Chi-square	1	5.727	< 0.05
5Females-NGA	Chi-square	1	7.875	< 0.01

* All tested insects responded

WR latency (Fig. 7B)
Factor: Effect of treatment on WR latency

Time after isolation	Statistical test	df	F value	*p*	Post hoc test	Pairwise comparison	*p*
2 hrs*	ANOVA	2	6.546	0.00438	Tukey's HSD	Control-GA vs Control-NGA	0.0878
						5Females-GA vs Control-NGA	0.3040
						5Females-GA vs Control-GA	0.0043
26 hrs	ANOVA	3	2.016	0.126	Tukey's HSD	Control-GA vs Control-NGA	0.5598
						5Females-GA vs Control-NGA	0.7104
						5Females-NGA vs Control-NGA	0.7039
						5Females-GA vs Control-GA	0.9933
						5Females-NGA vs Control NGA	0.1359
						5Females-NGA vs 5Females-GA	0.1996

* 5Females-NGA at 2 hrs: all insects did not respond

Factor: Recovery of WR latency (2 hrs, 26 hrs after isolation)

Treatment	Statistical test	df	T value	*p*
Control-GA	Unpaired Student's *t*-test	28	1.3581	0.1853
Control-NGA	Unpaired Student's *t*-test	20	-0.3471	0.7321
5Females-GA	Unpaired Student's *t*-test	20	-2.6187	0.0164
5Females-NGA*				

* 5Females-NGA at 2 hrs: all insects did not respond

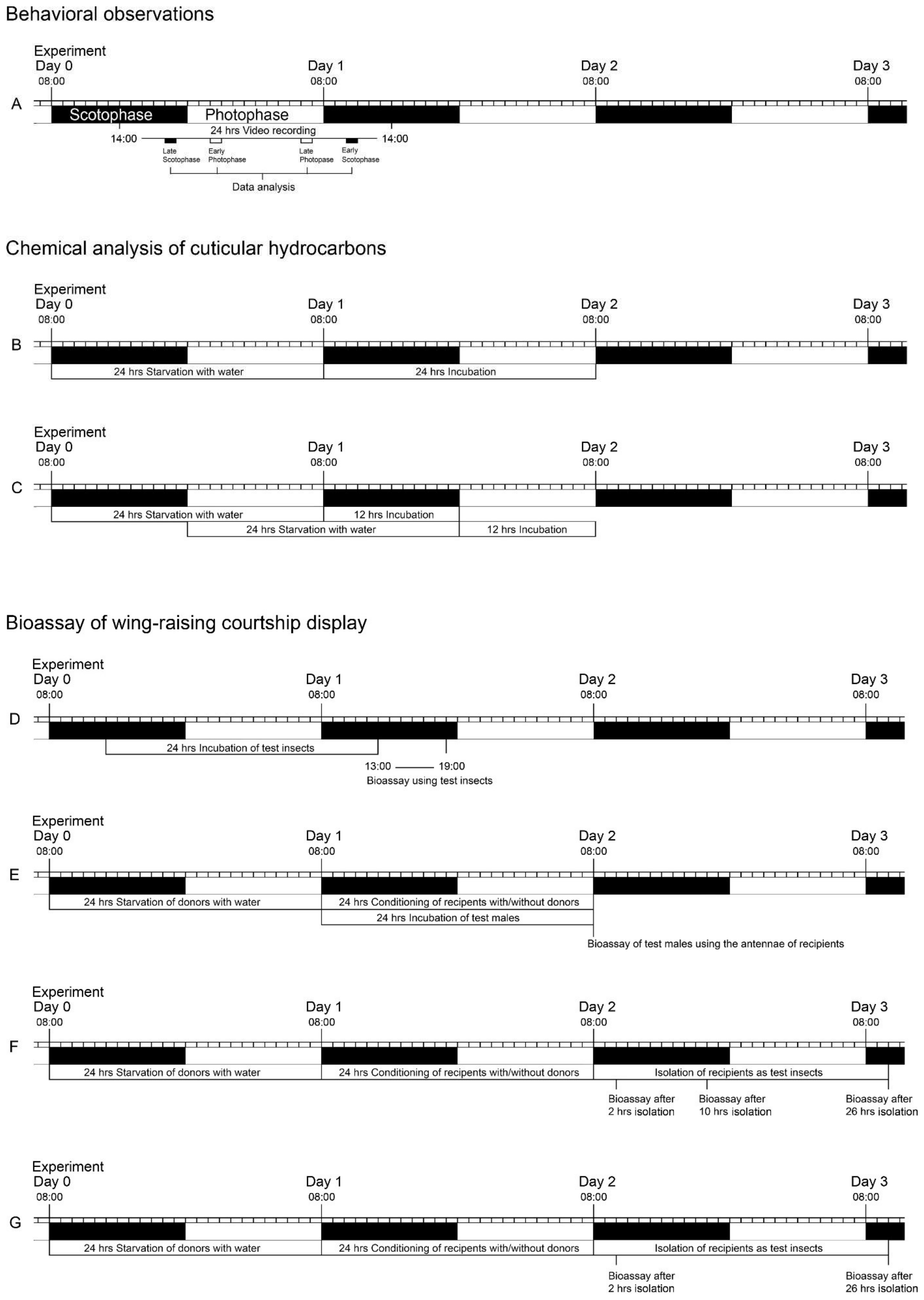
Behavioral observations
Experiment
Day 0
08:00
Day 1
08:00
Day 2
08:00
Day 3
08:00
A
Scotophase
Photophase
24 hrs Video recording
14:00
14:00
Late Scotophase
Early Photophase
Late Photopase
Early Scotophase
Data analysis
Chemical analysis of cuticular hydrocarbons
B
24 hrs Starvation with water
24 hrs Incubation
C
24 hrs Starvation with water
12 hrs Incubation
24 hrs Starvation with water
12 hrs Incubation
Bioassay of wing-raising courtship display
D
24 hrs Incubation of test insects
13:00 — 19:00
Bioassay using test insects
E
24 hrs Starvation of donors with water
24 hrs Conditioning of recipents with/without donors
24 hrs Incubation of test males
Bioassay of test males using the antennae of recipients
F
24 hrs Starvation of donors with water
24 hrs Conditioning of recipents with/without donors
Isolation of recipients as test insects
Bioassay after 2 hrs isolation
Bioassay after 10 hrs isolation
Bioassay after 26 hrs isolation
G
24 hrs Starvation of donors with water
24 hrs Conditioning of recipents with/without donors
Isolation of recipients as test insects
Bioassay after 2 hrs isolation
Bioassay after 26 hrs isolation

SI Figure 1. Time-course of various experiments.

A, Locomotor activity and grooming frequency for Fig. 1. B and C, Chemical analysis of cuticular hydrocarbons (CHCs) for Figs. 2 and 3. D–F, Bioassays of wing-raising displays for Fig. 4–7. The purpose of the 24 hrs starvation period applied to donors in E–F (experimental Day0 to Day1) is to allow the donor insects to excrete feces during the starvation period, to prevent the recipients from receiving fecal contamination from the donors during the 24 hrs of co-habitation (conditioning; Day1 to Day2).

SI Video 1. Context of the wing-raising (WR) display of male *B. germanica*.

The video shows the WR response of a male to an isolated female antenna in the “antenna-on-a-stick” assay. The male shows WR responses upon stimulation with only a female antenna, because a contact sex pheromone is contained in the female’s cuticular lipids.

Reference

Jurenka, R. A., Schal, C., Burns, E., Chase, J. & Blomquist, G. J. Structural correlation between the cuticular hydrocarbons and the female contact sex pheromone of the German cockroach *Blattella germanica* (L.). *Journal of Chemical Ecology* 15: 939-949, doi: 10.1007/BF01015189 (1989)
